# Supplementary material for: Integrative analysis of yeast colony growth
Source: Commun Biol. 2024 Apr 29;7:511. doi: 10.1038/s42003-024-06218-1 (PMC11058853; doi:10.1038/s42003-024-06218-1)
Supplement: Supplementary file 2 — Supplementary Information [file 42003_2024_6218_MOESM2_ESM.pdf]

Supplementary information to:

Integrative analysis of yeast colony growth

Tünde Gaizer[1,*], János Juhász[1,2,*], Bíborka Pillér[1], Helga Szakadáti[1], Csaba I. Pongor[1], Attila Csikász-Nagy[1]

[1]*Pázmány Péter Catholic University, Faculty of Information Technology and Bionics, Budapest, Hungary*

[2]*Semmelweis University, Institute of Medical Microbiology, Budapest, Hungary*

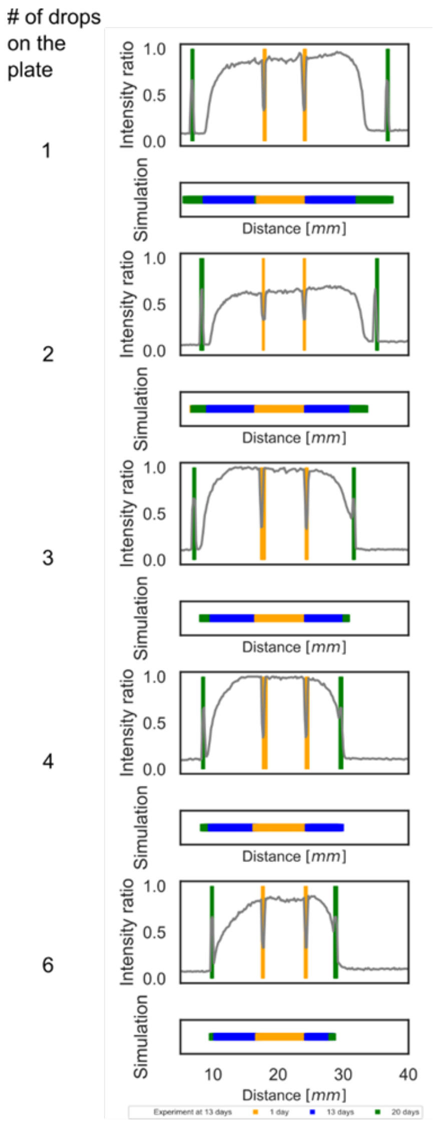

Supplementary Figure 1 Cross sectional intensity profiles of varying colony densities. Asymmetric growth is illustrated with the radial intensity profiles of the selected colonies. Left side is the edge of the Petri dish, righthand side is the middle of the dish. Colors denote the age of the colonies on the plate (see legend for color code). Experimental colonies' cross-sectional intensity ratios are shown at 13 days on the y-axis. Asymmetric growth can be observed in the 1 and 2 colonies cases the growth from 13 to 20 days towards the center of the dish (righthand side), the 3 and 4 colonies plates only grew outside in this period, while 6 colonies case did not show any expansion in this period. These results are resembled by the quantification of simulation results, showed as horizontal color bars for each panel.

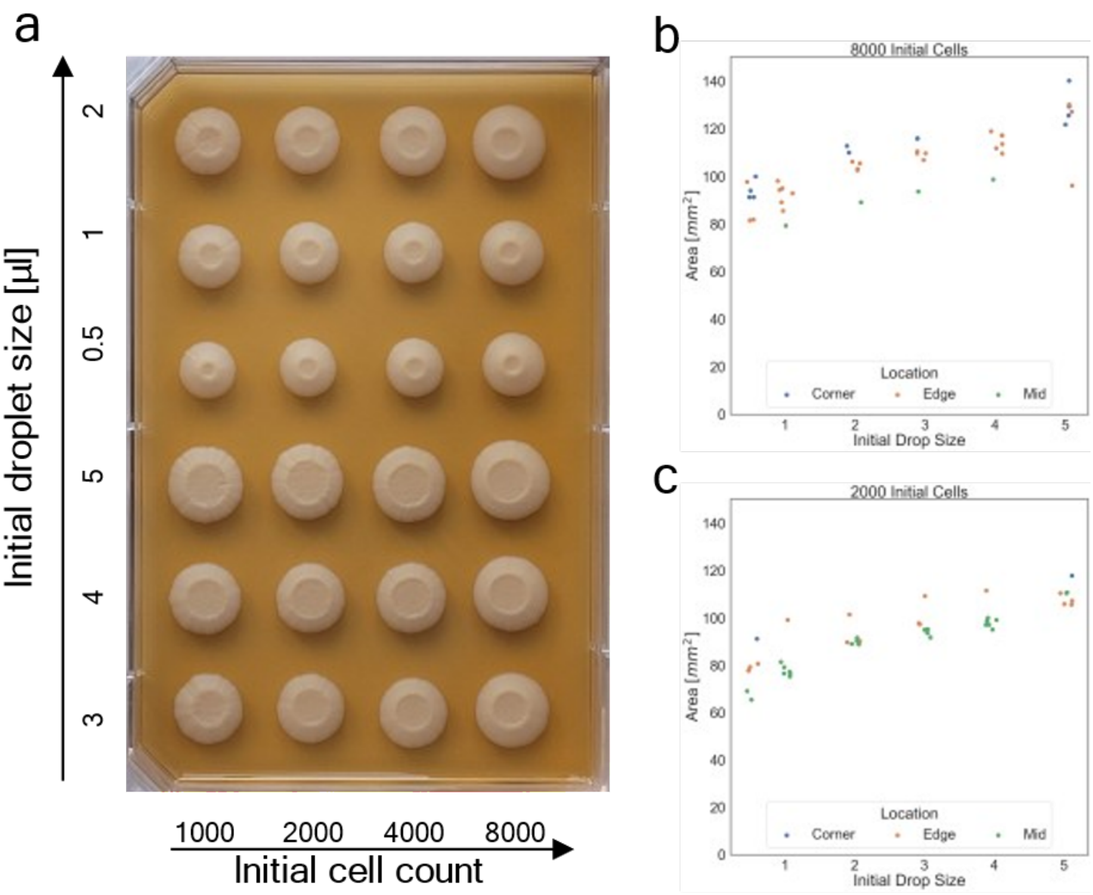

Supplementary Figure 2 Demonstration of edge effect Edge effect is demonstrated with a different layout at 12 days (a). Colony areas by pairs of initial cell counts (8000 cells in b and 2000 cells in c) and droplet sizes. Both highlighted examples show that cells on the edge or in the corner grew faster than colonies in the middle with the same initial condition.

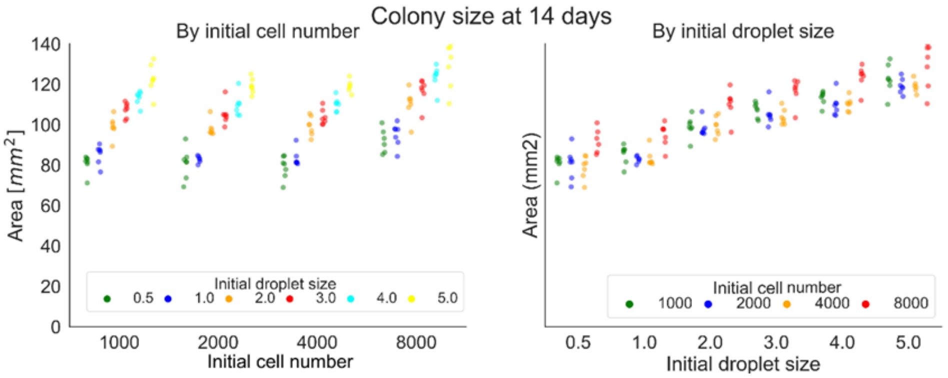

Supplementary Figure 3 Impact of the inoculation condition on the colony size at 14 days Area is shown by initial cell number and droplet size in both orders. While increasing trend can be observed for Initial droplet size, it is nearly flat for initial cell count. Colonies started from 8000 cells (most of which were on the edge of the plate) stand out from this trend.

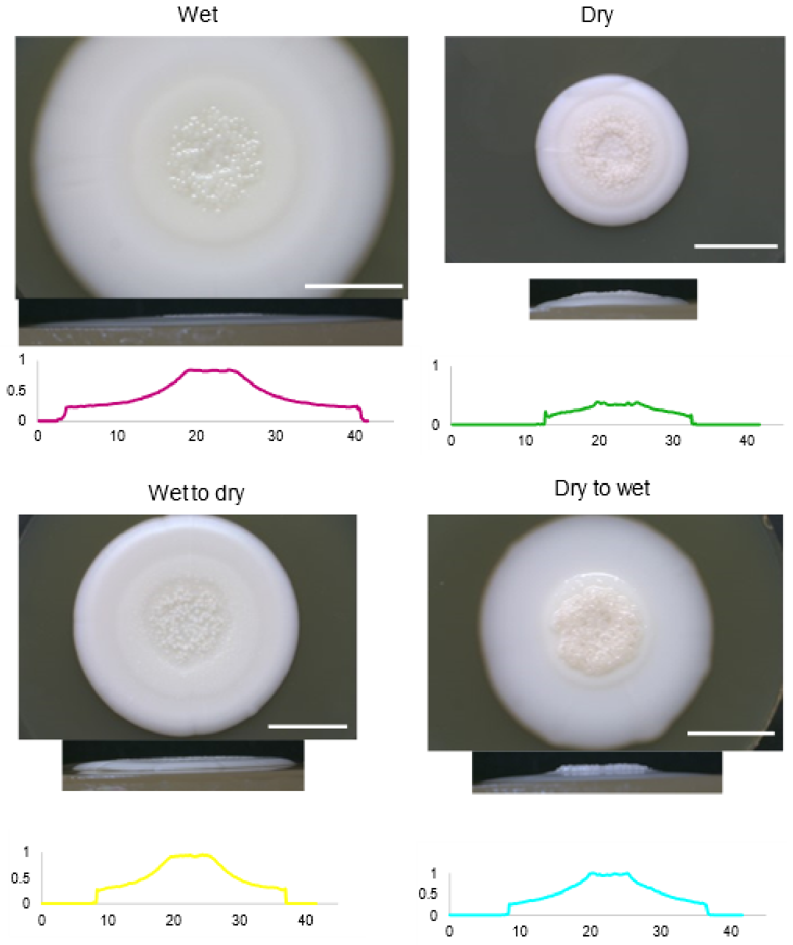

Supplementary Figure 4 Structure of colonies in wet and dry conditions. Colonies after 14 days display differing size and structure depending on the environmental conditions, they were grown in. Top and side view are shown for each condition. Pictures were taken with a stereomicroscope. Scale bar represents 10 mm.

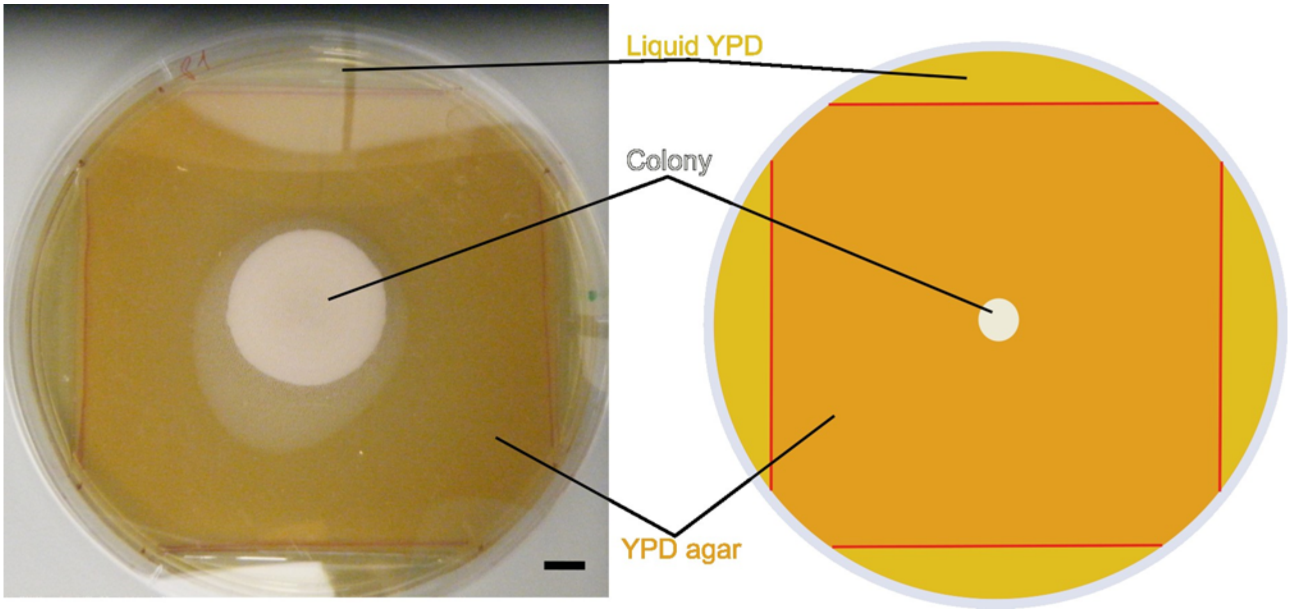

Supplementary Figure 5 Experimental set-up for growing giant colony. The edges of the plate were cut at the red lines after the YPD agar had solidified. Liquid YPD was regularly added to all the 4 slots on the edges. Scale bar represents 10 mm.

	Dry			Dry to wet			Wet to dry			Wet		
	**A1**	**A2**	**A3**	**B1**	**B2**	**B3**	**C1**	**C2**	**C3**	**D1**	**D2**	**D3**
**First phase average growth**	0.4352	0.4421	0.4584	0.4536	0.4607	0.4484	1.1188	1.1082	1.1248	1.0652	1.0582	1.0680
St error	0.04	0.00	0.01	0.01	0.02	0.01	0.02	0.04	0.04	0.03	0.02	0.03
r2	0.99	1.00	1.00	1.00	0.99	1.00	1.00	1.00	1.00	1.00	1.00	1.00
**Second phase average growth**	0.4011	0.3878	0.3073	1.8832	0.9687	0.9441	0.2835	0.3163	0.3553	0.9747	0.9854	0.9521
St error	0.02	0.03	0.02	0.50	0.02	0.04	0.02	0.02	0.02	0.04	0.04	0.02
r2	1.00	0.99	0.99	0.87	1.00	1.00	0.99	0.99	0.99	1.00	1.00	1.00

Supplementary Table 1: Average radial growth rates [mm/day] by sample derived from fitting a linear regression on the equivalent radius of each colony. The second phase growth rates were calculated from the 8th day only, allowing time for adjustment.

Supplementary Table 2: Input parameters and output descriptions of the model. Green: temporal parameters; yellow: spatial parameters of the environment or the agent; red: parameters defining material distribution in the environment; blue: cell metabolism and energetics related parameters; bold: fitted or fine-tuned parameters. Input parameters are defined in a .csv file. The simulations produce outputs for on-the-fly data visualizations, but also save .mat and .tsv data files for post processing.

Parameter name	Used value(s)	Description
**General and visualization parameters**		
Iteration number*	228 - 2160	length of the simulation (in simulation steps) 1 step~1 hour with the default settings
Time step	1	time resolution
Draw	0,1	1: visualizations during the simulation are on<br>0: no visualizations during the simulation
Visualization steps	20	frequency of updating the graphical output during the simulation (x indicates update in every xth steps)
**Environmental (plate, agar and nutrient related) parameters**		
Plate x*	620 - 900	x dimension of the plate (in grid cells)
Plate y*	620 - 900	y dimension of the plate (in grid cells) 1 grid cell ~ 55.6 mm^2 with default settings
Boundary condition	1	0: periodic boundary condition<br>1: fixed boundary condition (used in the presented work)
**Sigma1***	**30 – 80**	diffusion parameter of the top nutrient layer
**Sigma2***	**25 - 66.67**	diffusion parameter of the deep/lower nutrient layer
Flow rate	0.01	nutrient exchange rate between the two nutrient layers
**Initial nutrient level1***	**52 - 70**	initial nutrient content of each grid cell in the top layer (nutrient, energy and signal values are defined in abstract units)
**Initial nutrient level2***	**52 - 70**	initial nutrient content of each grid cell in the deep/lower layer
Sigma3	30	diffusion parameter of the signal
Decay rate	0.003	decay rate of the signal
**Cell (agent) parameters** (defined separately for each strain)		
Initial energy	3	initial energy content of each cell
Nutrient uptake	1.6	defines how much nutrient is taken up by an active cell in a simulation step
Nutrient uptake efficiency	1	1: all of the nutrient taken up is converted to energy in the cell, values between 0 and 1
Division threshold	5	active cells divide after reaching this energy threshold
**Division distance***	**0.63 – 1.38**	distance (in grid cells) between the daughter cell and its mother cell after division
Division distance decrease	inf	parameter for defining a time dependent decreasing (from 1 toward 0) multiplier of division distance. If the agar plate is drying with time, the colony growth slows down. It can be simulated via

		decreasing division distance. (This option was not used (was set to infinity) in the presented simulations.)
Metabolic energy	0.1	energy consumed by an active cell (from its internal energy pool) in each iteration
G0 threshold	0.3	active cells switch to stationary (G0) state if their energy levels fall below this threshold (this switch is reversible, if the energy level of a G0 cell exceeds this level, it switches back to active state)
G0 factor	0	multiplier for decreased nutrient uptake and metabolic energy on G0 state<br>0: G0 cells does not work (no energy uptake, metabolism)<br>1: no G0 effect
Death threshold	0.3	cells die if their energy levels fall below this threshold (their remaining energy returns to the top layer as nutrient)
Signal production	0	amount of signal produced by each active cell in every simulation step. Diverse signal effects can be specified, for example signal can control colony growth if it kills the cells or pushes them into G0 state above a certain threshold. (There is no signal production in the presented simulations.)
G0 signal production	0	multiplier (between 0 and 1) for signal production of G0 cells<br>0: no signal production in G0<br>1: no signal production decrease in G0
Signal effect1	80	the signal concentration affects the cells based on a sigmoid function. This parameter defines the change rate (slope) of the signal effect characteristics
Signal effect2	1	this parameter defines the signal value with 50% of the maximal signal effect (sets the range of effective signal concentrations)
Growth type	0	0: yeast-like growth<br>1: for filamentous growth: daughter cells stay close to the mother cell and cell lineages are forming a line of cells, via letting usually only the last cell to divide. (All simulations use yeast-like growth in the presented work.)
Division direction deviation	0	it defines the mean deviation of daughter cells from their mother cells in filamentous growth (0 for yeast-like growth), (it is needed for tortuous (but not random walk shaped) filaments, e.g.: 16 indicates pi/16 radian mean deviation from the division direction of the mother cell)
Branching probability	0	probability of an inner cell in a filament for cell division (results in the emergence of branched filaments)
Initial cell number*	1 - 800	number of cells of a certain type (yeast strain) at the beginning of the simulation
**Initial cell deviation***	**10 - 25**	radius (in grid cells) of the initial circular region (drop on the agar plate) populated with cells
Initial cell distribution	0.5	distribution of cells inside the initial drop (values e.g.: 0.5: uniform distribution; 0.25: more cells around the edge of the region; 1: more cell around the center of the region)
Initial drop center x	variable **	x coordinates (in grid cells) of the initial colony centers
Initial drop center y	variable **	y coordinates (in grid cells) of the initial colony centers

* See Supplementary Table 3 for the specific values in each simulation.

** See the configuration files for the exact coordinates at

https://github.com/CsikaszNagyLab/yeast_colony_growth_model .

The following information is gathered and stored about the *in-silico* experiments:

1. Agent numbers for each strain, for each metabolic state, and colony areas in every simulation step. Area is defined as the number of layer grid cells containing agents of a specific type. Area previously occupied by agents that are already dead is considered part of the colony area.
2. Data stored about the dead cells: their strain ID, position, and time (simulation step) of death.
3. Data stored about agents of the final colony: their strain ID, position, energy level, and metabolic state (active or G0)

Supplementary Table 3: Parameter modifications between the simulations. Green: temporal parameters; yellow: spatial parameters of the environment or the agent; red: parameters defining material distribution in the environment; bold: fitted or fine-tuned values; italic: values calculated based on the setups of the wet lab experiments. See Supplementary Table 2 for the other parameters and for their descriptions.

	**Simulation**							
**Parameter name**	**Fig.2 (1-6 colonies)**	**Fig.3b (24 colonies)**	**Fig.5 (unequal nutrient supply)**	**Fig.4a (dry condition)**	**Fig.4a (wet condition)**	**Fig.1c (single cell, small, bigger inoculation)**	**Fig.7a (dot near the colony)**	**Fig.6b (giant colony)**
Initial nutrient level1	**58.5**	**70**	70 (350)	**52**	**52**	52	52	52
Initial nutrient level2	**58.5**	**70**	70 (350)	**52**	**52**	52	52	52
Sigma1	**46.8**	**30**	30	30	**80**	30	30	30
Sigma2	**39**	**25**	25	25	**66.67**	25	25	25
Division distance	**0.795**	**0.63**	0.63	0.63	**1.38**	0.63	0.63	0.63
Initial cell deviation	**23**	**10, 12, 17, 20, 23, 25**	20	20	**23**	**0, 12, 20**	20	20
Iteration number	*480*	*228*	*480*	*312*	*312*	*336*	*864*	*2160*
Plate size	*620 x 620*	*870 x 540*	*870 x 540*	*620 x 620*	*620 x 620*	*620 x 620*	*620 x 620*	*900x900*
Initial cell number	*300*	*100, 200, 400, 800*	*300*	*300*	*300*	*1, 100, 300*	*300*	*300*

Supplementary Table 4: Effect of agent numbers to the final colonies. Statistics of 14 day long simulations started from different (30, 300 (default), 3000) initial agent sizes. Agent metabolism parameters were scaled up 10 times in the 30 agent scenario and scaled down 10 times in the 3000 agent scenario. The results demonstrate that the 30 initial agent case results in smaller and more irregular colony size, but the 3000 initial agent case produce similar colony size and shape (slightly smoother) to the default 300 initial agent scenario, but with longer simulation time and higher memory requirement.

**Initial agent number**	**30**	**300**	**3000**
**Final agent number**	45513	596792	6216080
**Final agent number/initial agent number**	1517.1	1989.31	2072.03
**Final colony area (grid point)**	6434	16686	19346
**Simulation time (min)**	0.4	5.7	69.3
**Size of stored data output from a simulation (MB)**	10.6	123	1220
**Final colony figure**	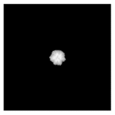	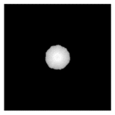	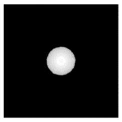
